# LEAP2: Next game-changer of pharmacotherapy for overweight and obesity?

**DOI:** 10.1016/j.xcrm.2022.100612

**Published:** 2022-04-19

**Authors:** Dan Liu, Sheyu Li

**Affiliations:** 1 Department of Endocrinology and Metabolism, West China Hospital, Sichuan University, Chengdu, Sichuan 610041, China

## Abstract

The world is seeking effective and safe weight-lowering drugs for centuries. LEAP2, acting on ghrelin and growth hormone secretagogue receptor, according to a study by Hageman et al.^1^ in this issue of *Cell Reports Medicine*, found its way toward the next target of pharmacotherapy for obesity and related diseases.

## Main text

Overweight and obesity and their related diseases are burdensome globally.^2^ The rapidly rising incidence warns the public that the world needs action. Bodyweight control requires long-term exercise and dietary regulation, but they are always challenging for many people who are overweight and obese, who may need additional help such as weight-lowering drugs. Ghrelin, known as the hormone of hunger, is among the culprits portraying dietary control as a nightmare for people suffering from overweight and obesity. As a brain-gut peptide, ghrelin originates from the stomach but acts on growth hormone secretagogue receptor (GHSR) in the brain and encourages food intake by stimulating pulsatile growth hormone secretion.^3^ Ever since its identification in 1999, accumulated works seek potential molecules that suppress the appetite in practice by blocking the activity of ghrelin as well as its downstream GHSR. Despite many ghrelin/GHSR-targeting agents like GHSR antagonists, GHSR inverse agonists, and GOAT (ghrelin O-acyltransferase; an enzyme essential for ghrelin activation) inhibitors have emerged, none have yet progressed to late-stage clinical trials for obesity or type 2 diabetes treatment due to the uncertainty of safety and/or effectivity in humans.^4^

Recent reports recognized liver-expressed antimicrobial peptide 2 (LEAP2), originally identified as an antimicrobial peptide, as a novel inverse agonist against GHSR constitutive activity and an endogenous competitive ghrelin antagonist.^5^ By inhibiting ghrelin and GHSR activation, LEAP2 potently blocks or blunts ghrelin-induced food intake, growth hormone release, and blood glucose elevation and receives negative feedback regulation.^5^^,^^6^ The plasma LEAP2 levels elevate in diet-induced obese mice and people with obesity compared with the lean controls.^7^ At the individual level, plasma LEAP2 concentration rises postprandially and falls during fasting within individuals.^5^ It is speculated that LEAP2 acts as a compensatory peptide against overeating and unhealthy weight gain. The mechanism and therapeutic potential of LEAP2 are clear but warrant proceeding in human trials.

Hagemann and colleagues, in their work published in the latest issues of the journal, moved one more step forward to the clinical use of LEAP2.^1^ Twenty healthy young males received continuous intravenous LEAP2 infusion or placebo in the randomized crossover trial, and those with LEAP2 intervention had suppressed *ad libitum* food intake and meal duration but not a liquid-mixed meal without additional hunger. The pilot observation did not identify any serious safety issues. Postprandial glucose and lipid profiles also improved. Nevertheless, not everybody responds well to the GHSR antagonist. The study further tested the LEAP2 in wild-type and GHSR-knockout mice and suggested the food intake suppression by LEAP2 highly relies on normal GHSR function. Interestingly, LEAP2 infusion suppresses postprandial growth hormone release but does not change ghrelin levels. The finding is in line with previous mechanical studies that indicated LEAP2 as a competitive antagonist of ghrelin. The study demonstrated a clear clinical potential of LEAP2 for people living with obesity and type 2 diabetes. Nevertheless, the benefits on body weight and glucose, as well as potential harms of LEAP2, warrant further exploration through larger trials in humans living with certain diseases.

Although the current preparation of LEAP2 is far from real-world practice, we could see its clear future. In Hagemann’s study, the half-life time of intravenous exogenous LEAP2 is as short as nine minutes on average, and the effective LEAP2 concentration is at least 2.6-fold higher than the physiological baseline. The fact calls for controlled or sustained-release preparation of LEAP2 that facilitates daily or weekly assumptions for real-world people living with obesity and type 2 diabetes. Fortunately, modern biotechniques and novel polymer materials made a buddle of good examples of long-acting peptides, including GLP-1 receptor agonists, which are among the most effective anti-diabetic and anti-obesity drugs worldwide.^8^^,^^9^ It is now the time for pharmacists to develop a safe preparation of LEAP2 with rigorous validation in preclinical and clinical studies. Another approach to translate LEAP2 into practice is to inhibit the key enzyme of its degradation by identifying small-molecule inhibitors. Nevertheless, the target remains unclear and needs further lab studies.^10^

In conclusion, Hagemann and colleagues moved another step forward toward the clinical pharmacotherapy targeting the ghrelin-GHSR pathway in people living with obesity, type 2 diabetes, and related diseases, but there is still a critical gap in the research that will require innovative biological, pharmaceutical, and clinical exploration. We look forward to seeing the path this research will take.
